# A new behavioral health services cascade framework for measuring unmet addiction health services needs and adolescent offenders: conceptual and measurement challenges

**DOI:** 10.1186/1940-0640-10-S1-A4

**Published:** 2015-02-20

**Authors:** Steven Belenko, Tisha Wiley, Danica Knight, Michael Dennis, Gail Wasserman, Faye Taxman

**Affiliations:** 1 Department of Criminal Justice, Temple University, Philadelphia, PA, 19122, USA; 2 Services Research Branch, National Institute on Drug Abuse, Rockville, MD, 20852, USA; 3 Institute of Behavioral Research, Texas Christian University, Fort Worth, TX, 76129, USA; 4 GAIN Coordinating Center, Chestnut Health Systems, Normal, IL, 61761, USA; 5 Columbia University, New York, NY, 10027, USA; 6 Criminology, Law and Society Department, George Mason University, Fairfax, VA, 22030, USA

## 

Juvenile Justice-Translational Research on Interventions for Adolescents in the Legal System (JJ-TRIALS) is a cooperative implementation science initiative launched by NIDA in July 2013. The project seeks to reduce unmet substance use disorder needs for delinquent youth under community supervision by assisting juvenile justice (JJ) agencies in their efforts to implement best practices and improve service utilization along a behavioral health services cascade (screening, assessment, referral, initiation into treatment, and retention in treatment). Although many youth under JJ supervision have substance use and associated mental health disorders, there are numerous gaps in the identification of these problems and referral to appropriate services. Linkages between the JJ and behavioral health systems can be problematic because of the lack of shared mission, limited training of JJ staff on behavioral health issues, and limited sharing of information across service systems. Ideally, JJ staff will screen and assess youth for substance use and related disorders, and refer them to appropriate evidence-based treatment when needed; the youth should initiate treatment and be retained for a clinically effective period. The ultimate goal is to maximize the proportion of substance-involved youth identified and retained in treatment. Based on the HIV services cascade, we developed a conceptual framework, the Behavioral Health Services Cascade. This framework provides a useful heuristic for visualizing and measuring movement through the continuum of services. It illustrates the level of unmet needs at different points and provides a measurement framework for tracking improvements in reducing unmet needs. The model helps agency staff and leadership and policymakers understand the sequential and related stages of the services continuum and identify stages/linkage points that may need improvements. In the main JJ-TRIALS protocol, we are using the cascade framework to: 1) monitor and evaluate the primary outcome of reducing unmet service needs; 2) use local data to populate the cascade and provide feedback to agencies for need assessments and site feedback reports; and 3) help site staff select and monitor strategic goals to reduce gaps in one or more component of the cascade. We will also be developing measures related to the quality of the services delivered at each point in the cascade. Some challenges being addressed include: 1) defining and collecting consistent measures across multiple states and counties; 2) inconsistency in data availability across sites, especially on treatment engagement and continuing care; and 3) accounting for multiple and sometimes nonlinear processes at different points in the cascade.

